# The Influence of Fluorine on the Disturbances of Homeostasis in the Central Nervous System

**DOI:** 10.1007/s12011-016-0871-4

**Published:** 2016-10-27

**Authors:** K. Dec, A. Łukomska, D. Maciejewska, K. Jakubczyk, I. Baranowska-Bosiacka, D. Chlubek, A. Wąsik, I. Gutowska

**Affiliations:** 1 Department of Biochemistry and Human Nutrition, Pomeranian Medical University in Szczecin, Broniewskiego street 24, 70-406 Szczecin, Poland; 2 Department of Biochemistry, Pomeranian Medical University in Szczecin, Powstańców Wlkp. 72 av., 71-111 Szczecin, Poland; 3 Institute of Pharmacology, Polish Academy of Sciences, Department of Neurochemistry, Smętna street 12, 31-343 Kraków, Poland

**Keywords:** Fluoride, Brain, Neurotoxicity, Neuroinflammation, Apoptosis, Prenatal exposition

## Abstract

Fluorides occur naturally in the environment, the daily exposure of human organism to fluorine mainly depends on the intake of this element with drinking water and it is connected with the geographical region. In some countries, we can observe the endemic fluorosis—the damage of hard and soft tissues caused by the excessive intake of fluorine. Recent studies showed that fluorine is toxic to the central nervous system (CNS). There are several known mechanisms which lead to structural brain damage caused by the excessive intake of fluorine. This element is able to cross the blood-brain barrier, and it accumulates in neurons affecting cytological changes, cell activity and ion transport (e.g. chlorine transport). Additionally, fluorine changes the concentration of non-enzymatic advanced glycation end products (AGEs), the metabolism of neurotransmitters (influencing mainly glutamatergic neurotransmission) and the energy metabolism of neurons by the impaired glucose transporter—GLUT1. It can also change activity and lead to dysfunction of important proteins which are part of the respiratory chain. Fluorine also affects oxidative stress, glial activation and inflammation in the CNS which leads to neurodegeneration. All of those changes lead to abnormal cell differentiation and the activation of apoptosis through the changes in the expression of neural cell adhesion molecules (NCAM), glial fibrillary acidic protein (GFAP), brain-derived neurotrophic factor (BDNF) and MAP kinases. Excessive exposure to this element can cause harmful effects such as permanent damage of all brain structures, impaired learning ability, memory dysfunction and behavioural problems. This paper provides an overview of the fluoride neurotoxicity in juveniles and adults.

## Introduction

Fluorine is an active non-metal that occurs in the environment and that is used in industry and medicine (diagnostics, prevention) [1]. The daily exposure of our organisms to fluorine mainly depends on the geographical region we inhabit. The most important factor contributing to the exposure is the content of fluorine in drinking water and, to a lesser extent, in air and food [2, 3]. Moreover, this element is commonly used in the prevention of dental caries due to its effectiveness and the low costs of manufacture of products for oral care [2, 4–6].

In the organisms of infants and children, about 80–90 % of the absorbed fluorine is accumulated. A smaller amount is stored in the organisms of adults (60 %). Of the received fluorine, 75 %–90 % undergoes absorption in the stomach and intestines, and 99 % of the fluorine that gets to the circulatory system is transported to tissues rich in calcium (mainly to hard tissues). Retrospective studies showed that the symptoms of fluorosis (the disorder of the physiology of bones and teeth and the damage to soft tissue) appeared when the supply of fluorine was over 0.15 mg/kg/24 h [2, 3, 6–9]. In recent years, scientists have been focusing on the toxic influence of this element on the nervous system. Prolonged exposure to fluorine in the prenatal and postnatal stages of development has a toxic influence on the metabolism and physiology of neurons and glia which results in disorders in the processes connected with memory and learning [4, 10, 11]. Epidemiological studies carried out in geographical regions in which fluorine content in drinking water is high showed that children who live in those areas have a statistically significant decreased level of intelligence in comparison to children from regions not contaminated with fluorine [10, 12, 13]. Fluorine exposure in the prenatal and neonatal periods is dangerous because this element has the ability to penetrate through the placenta and it is able to cross the blood-brain barrier. Young individuals are less resistant to the toxic influence of fluorine due to the fact that their defensive mechanisms are not fully developed and the permeability of their blood-brain barrier is higher than among adults [2, 14–16]. This phenomenon was confirmed by a research carried out on rats. The animals were exposed to high levels of fluorine (10, 25, 50 mg/L) for 8 months. The content of fluorides in the rats’ brains was even 250 times higher than in the control group [9]. However, the exact mechanisms by which fluorine decreases cognitive and learning abilities and causes memory loss were not clearly defined. So far, the element has been studied in terms of its influence on neurotransmission, the synthesis of proinflammatory factors, free-radical processes and the apoptosis of cells of the central nervous system [17].

### Cytological Changes within Neurons

Microtubules consisting of compact heterodimer tubulins form the cell cytoskeleton in which organelles are suspended. Depending on the type of cells, microtubules can reach the length of even a few millimetres, and their elasticity and the ability to adjust the length through building or degrading heterodimers are of particular importance for the physiology of cells [18]. A proper construction of the cytoskeleton is important for the functioning of neurons. It was observed that the disorders in the construction of microtubules influence the deterioration of dendrites, the degeneration of axons and the decrease in the number of Purkinje cells [9, 19]. Among adult mice exposed to fluorine, a decrease in the expression of tubulins forming the heterodimers (Tuba1 and TubB2a) in the hippocampus was observed (the content of fluorine in drinking water, 100 mg/L). The disorders in the synthesis of tubulins are important in relation to such processes as the maturing or the division of cells because they might lead to the creation of malfunctioning neurons without the ability of signal transmission [9].

The accumulation of fluorine in the brain also influences the content of Nissl bodies, which are concentrations of ribosomes and RNA in neurons. These concentrations are responsible for the characteristic colour of grey matter. Among adult rats exposed to relatively low concentrations of fluorine, a significant decrease of the content of this Nissl substance was observed (the concentration of fluorine in drinking water, 2.1 and 10 mg/L). These values are calculated in the active neurons. Their content decreases in cells that are growing old and degenerating [1].

### Neuron Activity and the Transportation of Ions

The research carried out on adult specimens showed a negative influence of fluorine on the volume of neurons. The regulation of the volume of cells and the concentration of ions have a significant influence on the preservation of homeostasis in the nervous system [4, 20]. The stimulation of a nervous synapse is accompanied by the increase of the cell’s volume by 4–30 % of the initial volume. Such changes influence neuron activity because they are related to the changes in the flow of ions and water from the cytoplasm to the extracellular space and vice versa [21–23]. Fluorine (5 mM) causes disorders in the homeostasis of neurons in the hippocampus of adult rats and mice by increasing the outflow of chloride ion from cells and by changing the activity of proteins from the MAP kinase family. This leads to the decrease of the volume and activity of neurons [4]. MAP kinases, i.e., mitogen-activated protein kinases, are a family of proteins that take part in the regulation of the response to extracellular factors such as mitogens. The proteins ERK and JNK and the isoforms of protein p58 belong to the family of serine-threonine kinases. These proteins influence the regulation of the growth and differentiation of cells, the regulation of apoptosis and the expression of genes [24]. However, recent analysis proved that they also take part in the regulation of the activity of membrane transporters. Fluorine, through its influence on the activity of Ras protein, activates a cascade of reactions that leads to the activation of ERK. This influences the membrane ion channels and leads to changes in the flow of ions (including an increased outflow of chloride ion) and in the nervous cell volume causing disorders in cell metabolism, in cell functioning and, most of all, in the transmission of nerve impulses [25, 26].

### The Energy Metabolism of Neurons

The activity of mitochondria is a very important factor which influences numerous processes and the lifespan of neurons. Due to their limited glycolytic capabilities, these cells depend on the processes of oxidative phosphorylation which is the main source of energy in the central nervous system. The energy created by mitochondria is used for the activity of membrane ion channels and for the transmission of impulses through synapses, and the dysfunction of these organelles is observed in neurodegenerative illnesses [27, 28]. One of the important factors influencing the energy metabolism of neurons is the transportation, absorption and transformation of glucose, because it serves as the main source of energy for neurons [29]. It is common knowledge that providing proper amounts of glucose to an organism significantly influences the improvement of cognitive functions, and numerous analyses confirmed that disorders in glucose metabolism may be the cause of the death of neurons [30–32]. Among rats exposed to fluorine, decreased glucose usage was observed (the concentration of fluorine in drinking water, 50 and 100 mg/L) as well as a decrease in the expression of the main receptor responsible for the glucose uptake in the nervous system, GLUT1 (the concentration of fluorine in drinking water, 25, 50 and 100 mg/L), in the cerebral cortex and hippocampus [30].

An equally important factor for the functioning of mitochondria is oxidative stress. Oxidative stress caused by the excessive production of reactive oxygen species (ROS) significantly influences the functioning of neurons through its influence on the activity of mitochondrial enzymes. Increased ROS synthesis in the mitochondria of nervous cells, disorders in integrity and changes in the potential of mitochondrial membrane were observed among rats which received fluorine in the proportion of 13 mg/kg/24 h [33]. Prenatal and postnatal exposure of mice to high concentrations of fluorine in drinking water (150 mg/L) caused disorders in the energy metabolism of the cerebral cortex of the animals. One of the observed facts was the increased activity of one of the subunits of ATP synthase—ATP5h. This enzyme consists of several subunits that form two dimers—F1 and F0. F1 is the catalytic part, whereas F0 is a membrane subunit which takes part in the transportation of protons. The disorders in the functioning of these subunits lead to changes in the energy balance (ATP/ADP) within the cell. The analysis showed the hyperactivity of subunit ATP5h, a part of the content of F0, which leads to disorders in the activity of ATP synthase [15, 34]. The exposure to fluorine also caused a decrease in the activity of NADH-ubichinon oxidoreductase which is a part of respiratory chain complex 1. It is involved in the transportation of ions in the chain and in the creation of sodium gradient necessary in the process of ATP synthesis. The aforementioned changes in the activity of ATP synthase and NADH-ubichinon oxidase lead to a significant decrease of ATP synthesis in the mitochondria of nervous cells [15, 35]. Another study carried out on mice confirmed that fluorine influences the activity of complexes of the respiratory chain and of enzymes of the citric acid cycle. A decrease in the activity of complexes I, II, III and IV; isocitrate dehydrogenase and succinate dehydrogenase was observed in the cerebral cortex, cerebellum and hippocampus of mice exposed to high concentrations of fluorine (the concentration of fluorine in drinking water, 270 mg/L) [27].

A decrease in the activity of the respiratory chain complexes influences the increase in the synthesis of ROS which activate the pathways leading to the degradation of mitochondria as well as the entire cell [27]. ROS and the products of lipid peroxidation are also responsible for the formation of compounds that block the active area of isocitrate dehydrogenase, consequently inhibiting the oxidative decarboxylation of the isocitrate in the Krebs cycle and influencing the activity of this pathway. Fluorine itself influences the activity of many enzymes through its ability to break the hydrogen bonds in proteins—e.g. in the enzyme active centre [36, 37]. Furthermore, the increase in the synthesis of free radicals in mitochondria leads to the initiation of oxidative stress and the degradation of mitochondrial DNA. The result of these processes forms another phenomenon that takes place in the mitochondria—the disorder of the expression of enzymes necessary for the synthesis of ATP and the decrease of ATP concentration. This, in consequence, leads to the activation of the processes that cause the death of the cell [33].

### Oxidative Stress and the Activity of Anti-Oxidative Enzymes

The analysis carried out with the usage of experimental animal models more than once confirmed that the accumulation of fluorine in the central nervous system initiates inflammatory and degenerative processes through the activation of oxidative stress in both young and adult specimens. Oxidative stress is caused by the disturbance in the balance between the synthesis of ROS and the activity of anti-oxidative enzymes. The increasing concentration of ROS leads to metabolism disorders, the initiation of inflammatory states and the disorders of the differentiation, maturing and division of cells. This, in consequence, causes tissue damage [17, 38]. ROS do not only cause disorders in the signal pathways of cells, but if their concentration is high, membrane lipid release and oxidation take place. The products of this process might be further transformed into physiological and pharmacological active inflammatory compounds [39].

Numerous analyses carried out on cell cultures and animal models confirmed that fluorine accumulation in the brain leads to the increase of the concentration of ROS, the decrease of the activity of antioxidative enzymes and the increase of the intensity of lipid peroxidation. In the neuron cultures isolated from the hippocampus, after 48 h of fluorine incubation (concentration, 40 and 80 mg/L), several phenomena were observed: the increase in the synthesis of ROS and the derivatives of lipid peroxidation, malondialdehyde (MDA), the decrease of the activity of antioxidative enzymes, superoxide dismutase (SOD) and glutathione peroxidise (GPx), and the decrease of the concentration of glutathione [40]. Increased activity of catalase (CAT) was observed among young rats exposed to fluorine, which might point to the activation of protective mechanisms against the harmful activity of oxygen free radicals in the organism (the concentration of fluorine in drinking water, 30 and 100 mg/L) [33, 41]. In relation to adult rats that received 20 mg of fluorine per kilogram of body mass every 24 h, it was observed that the concentration of glutathione in the brain decreased, the production of the radicals OH and NO increased and the activity of antioxidative enzymes CAT, SOD, GPX and glutathione reductase (GR) was smaller [5]. Moreover, increased intensity of oxidation of lipids and proteins was observed in the cerebral cortex, cerebellum and medulla oblongata (the concentration of fluorine in drinking water, 50 and 150 mg/L). These results were confirmed by another analysis in which, among rats exposed to fluorine in the prenatal and postnatal periods, increased concentrations of fluorine in the serum and the brain were observed as early as on the 14th day of life, which resulted in the decreased activity of SOD and higher intensity of lipid peroxidation in the brains of the analysed animals (the concentration of fluorine in drinking water, 20 mg/L) [33, 42]. An increase in the concentration of the products of lipid oxidation was also observed among adult rats that were exposed to 10 mg/L of fluorine concentration in drinking water. In the case of these animals, SOD activity was also smaller [17].

The analyses carried out so far show that one of the mechanisms by which fluorine influences the disorders in brain functioning is the increase of the synthesis of ROS and the weakening of the defensive mechanisms against their activity (the decrease in the activity of antioxidative enzymes) [43]. In the central nervous system, the intensity of the processes that utilize oxygen is very high. Moreover, there are high concentrations of easily oxidizable fatty acids, and the activity of antioxidative enzymes is relatively small in comparison to other tissues [7, 44]. Long-lasting oxidative stress causes the “wear” of enzymes responsible for the removal of free radicals. Their increasing concentration in cells causes lipid peroxidation and the oxidation of proteins and nucleic acids [40, 45]. The changes in the content of membrane phospholipids in neurons, caused by their release and oxidation, result in the changes in the fluidity, stability and permeability of the cell membrane [46, 47]. Furthermore, by activating different signal paths, oxidative stress leads to the decrease in the lifespan of cells, the disorders in growth and differentiation and the initiation of apoptosis [7, 48, 49].

### The Advanced Glycation End Products

The advanced glycation end products (AGEs) of proteins and lipids are created spontaneously in living organisms in a multi-stage process that does not undergo enzymatic catalysis. In physiological conditions, AGEs fulfil regulatory functions, including the inhibition of cell differentiation. A high content of glycation products was observed in foetal stem cells. Their intensive degradation ensues when they begin to differentiate. The increase in the synthesis and the concentration of AGEs in the organism is observed in pathological states [43, 50]. In properly functioning organisms, AGEs are quickly degraded in proteasomes. However, in some products, cross-linking occurs, which influences the physicochemical properties of these cells and leads to the creation of insoluble aggregates. AGEs are bound by specific receptors. Currently, there are five known types of receptors that bind the products of non-enzymatic glycation: MSR-1 (macrophage scavenger receptor), AGE-R1, AGE-R2 (which binds phosphoproteins), AGE-R3 (which recognizes galactosidic groups) and RAGE (the receptor for AGEs). While the receptors AGE-R1–3 and MSR-1 take part in the removal of the products of glycation from circulation, RAGE acts as a signal receptor activating the processes connected with the synthesis of ROS, transcription factors and the proinflammatory particles such as nuclear factor kB (NF-kB) and the previously mentioned MAP kinases (MAPK) [43, 50, 51]. RAGE demonstrates expression on macrophages, T lymphocytes, cardiomyocytes, endothelial cells, smooth muscle cells, neurons and dendritic cells [52]. The accumulation of AGEs in neurons increases the synthesis of ROS, disturbs the transmission of nervous impulses and stimulates the atrophy of nerve fibres. Moreover, it correlates with the increased expression of proteins that regulate the apoptosis and differentiation of cells [43, 53].

In vivo research carried out on adult rats showed that fluorine increases the concentration of products of advanced glycation of proteins in cells (the concentration in drinking water, 50 mg/L) [43]. A significant increase in the expression of RAGE and NADPH oxidase 2 (NOX2) was also observed among specimens exposed to fluorine for 6 months (the concentration in drinking water, 5 and 50 mg/L). In order to determine the influence of NOX2 on the activation of AGE/RAGE, simultaneous research with cell cultures was carried out. SH-SY5Y cells originating from human neuroblastoma were incubated with various concentrations of fluorine for 48 h (the applied concentrations, 0.5, 5 and 50 mg/L). The following phenomena were observed in the culture with the concentration of 50 mg/L: a significant increase in ROS and MDA after 6 h of incubation, increased expression of NOX2 after 12 h of incubation and RAGE after 24 h of incubation and an increased concentration of AGE in cells after 36 h of incubation (the concentrations of the analysed particles positively correlated with the time of incubation). The analysis confirmed that one of the mechanisms that lead to the degeneration of neurons is the activation of the AGE/RAGE complex. An additional analysis carried out in vitro confirmed that one of the mechanisms that lead to the activation of AGE/RAGE is the increase in the activity of NOX2 [43]. An increased intensity of non-enzymatic glycation of proteins is predominantly observed when the activity of oxidative factors becomes stronger and, at the same time, when the antioxidative mechanisms are inhibited. As mentioned before, fluorine strengthens the synthesis of free radicals and decreases the activity of antioxidative enzymes in the central nervous system which may strengthen the synthesis of AGEs and the expression of RAGE on the membranes of neurons. This, in consequence, may cause pathological accumulation of AGEs in the cells of the nervous system [43, 52]. The accumulation of AGEs in neurons activates proinflammatory transcription factors—NF-kB initiating the inflammatory state and MAP kinases which change the permeability of the membrane of nerve cells and may activate the apoptosis pathway. These processes may initiate the demyelination of dendrites and the degeneration of neurons.

### The Synthesis of Proinflammatory Factors

One of the factors indicating the initiation and development of an inflammatory state in all organs is the change in the concentration of interleukins. Interleukins belong to cytokines—proteins that take part in the intercellular signalling and the regulation of immunological response [54]. Cytokines in the nervous system influence the regulation of sleep and the processes connected to memorizing. They also take part in neurodegenerative processes, and they help keep the integrity of the blood/brain barrier [55]. Interleukin 6 (Il-6) is secreted by macrophages, B-lymphocytes, microglia, neurons, adipocytes, myocytes and fibroblasts, but only a few cells show the expression of receptors for Il-6—some of the leukocytes and microglial cells. In the nervous system, astrocytes and oligodendrocytes do not show the expression of receptors for Il-6 [54]. In physiological conditions, a low concentration of Il-6 in the nervous system is observed [56]. In low concentrations, Il-6 may have a neuroprotective effect—it influences the differentiation of oligodendrocytes, acts as a neurotrophic factor and takes part in the regeneration of neurons and, in relation to mice among which the Il-6 gene is blocked, the activation of microglia is decreased. The rise in the concentration of Il-6 appears in inflammatory states and neurodegenerative illnesses [57, 58]. Among illnesses with an inflammatory basis and neurodegenerative diseases, an increased concentration of other cytokines such as Il-1B and TNF-α is also observed. These cytokines are responsible for the initiation of inflammatory states and the death of neurons [59]. Research shows that the exposure of adult rats to fluorine causes the activation of microglia in the hippocampus and cerebral cortex and that it initiates an inflammatory state through the synthesis of proinflammatory cytokines—Il-1B, Il-6 and TNF-α (the concentration in drinking water, 60 and 120 mg/L) [60].

### The Activation of Glial Cells and the Migration of Lymphocytes to CNS

Despite the big numbers of glial cells located in the nervous system (it is estimated that there are 10 times more of these cells than neurons), they do not take part in the information transfer, but their task is to support the activity of neurons. We can divide them into macroglia that include astrocytes, oligodendrocytes and Schwann cells, and microglia. Astrocytes fulfil functions crucial to maintaining the homeostasis and the proper functioning of the nervous system. Among other roles, they are responsible for the metabolism and the preservation of the proper concentration of potassium, the regulation of pH, the metabolism and transport of neurotransmitters and the regulation of the strength of stimulation [17, 61]. The rise in the number of glial fibrillary acidic protein (GFAP) cells indicates the activation of astrocytes and the initiation of defensive mechanisms against harmful factors, such as fluorine. GFAP is a protein specific for astrocytes. Its expression increases when the cells of the nervous system become damaged or when they show disorders in metabolism. It stimulates their proliferation in order to minimize and repair the damage [17, 62]. Microglia are cells differentiating from macrophages, which are “settled” in the nervous system. As in the case with astrocytes, their task is to preserve the balance in the nervous system. However, their influence and activity are homological to the activity of macrophages. They are responsible for the absorption of the products of nerve tissue breakdown. Therefore, their proliferation is stimulated by external factors such as improper proteins that find their way into intercellular space or substances released from dying neurons [63]. The activation of microglia might occur when the pathway associated with the activity of MAP–ERK kinase is initiated. Oxidative stress in the nervous system causes an increase in the expression of ERK in glial cells which initiates the synthesis of proinflammatory substances [64, 65]. The migration of B lymphocytes to the nervous system is observed in pathological states. They take part in the inhibition of damage progression and in the repair processes engaging, among others, microglia [17, 34].

Immunohistochemical analyses carried out on the material collected from adult rats exposed to fluorine showed that the activation of astrocytes takes place in the cerebral cortex (the concentration of fluorine in drinking water, 10 mg/L). In the studied material, an increased immunoreactivity of GFAP was observed, which is specifically connected to astrocytes. The research also confirmed that the activation of microglia and the migration of B cells took place in the brains of the exposed rats. The research consisted of antibodies anti-CD68 which are specific to cells originating from the line of macrophages, including microglia, and of antibodies anti-CD20 specific to B cells [17].

### The Metabolism of Neurotransmitters

Another mechanism by which fluorine may influence the disorders in the functioning of neurons is the change in the concentration of neurotransmitters. Prolonged exposure to fluorine causes a decrease in the concentration of glutamate in the brain. Glutamate constitutes about 30 % of all neurotransmitters in the central nervous system and is the main stimulating transmitter. It is secreted in high amounts in the hippocampus, which is responsible for the processes of memorizing and learning [66–68]. This amino acid is supplied to the organism with the diet. However, only a small amount of it passes through the blood/brain barrier. This is a protective mechanism against excessive inflow of this neurotransmitter to the brain which could cause the depolarization and damaging of neurons [69, 70]. Therefore, in the central nervous system, there has to be a balance between the synthesis of the endogenic glutamate and its loss. In the synthesis of glutamate, aspartate transaminase (AST) and alanine aminotransferase (ALT) take part. Their activity is inhibited by an excessive supply of fluorine [69, 71]. This element also increases the activity of glutamate decarboxylase (GAD) which results in the transformation of glutamate into γ-aminobutyric acid (GABA)—the main inhibiting transmitter in the nervous system. These processes lead to the decrease in the pool of glutamate in the brain leading to dysfunctions in synaptic transmission and the disorders in cognitive functions.

Bergmann glial cells (BGC) are abundantly present in the cerebellum. They participate in the transportation and metabolism of neurotransmitters, the preservation of the balance of potassium and the correct pH in the nervous system. One of the more important functions of BGC is the metabolism of glutamate. These cells are one of the main sources of this neurotransmitter, and to a large extent, they are responsible for glutamatergic stimulation [6, 72]. The incubation of BGC cells (isolated from the cerebellum of rats) with fluorine in concentrations of 1 and 2 mM caused a significant decrease in the lifespan of cells, causing disorders in the metabolism of glutamate [6, 73]. Other research showed a decrease in the concentration of glutamate in the serum, hippocampus and cerebral cortex of the studied animals (the concentration of fluorine in drinking water, 120 mg/L), an increase in the activity of glutamic acid decarboxylase and a decrease in the activity of asparagine transferase and alanine transferase—the enzymes participating in the metabolism of glutamate (the concentration of fluorine in drinking water, 150 mg/L) [66, 69, 74]. Glutamate participates in memory processes through the stimulation of specific ionotropic and metabotropic (mGluRs) receptors. One of the things that the receptors of the mGluR I group (mGluR1 and mGluR5) are responsible for is the preservation of the correct plasticity of synapses. They show expression in the hippocampus, cerebral cortex and cerebellum. The inhibition of the expression of mGluR5 among test animals causes significant disorders in spatial orientation and “spatial learning”. Fluorine causes an insignificant decrease in the expression of mGluR5 [66, 75]. Glutamatergic stimulation is strongly related to memory (mainly long-term memory) and learning, so all the disturbances of the synthesis and transportation of glutamate negatively influence those two processes [6, 76].

Fluorine also causes changes in the secretion of neurotransmitters such as serotonin, dopamine, norepinephrine, acetylcholine and epinephrine (the concentration of fluorine in drinking water, 20, 40 and 60 mg/L) [77]. Adult rats that received drinking water with 100 mg/L concentration of fluorine had a higher concentration of noradrenalin and serotonin in the hippocampus, striatum and cerebral cortex [78].

### The Expression of Proteins that Regulate the Maturing, Differentiation and Proliferation of Neurons

Constant exposure of young specimens to fluorine in the prenatal and postnatal periods initiates processes that lead to the degeneration of neurons through the influence on the expression of regulatory proteins. Among other phenomena, changes in the demyelinating character, a decrease in the number of Purkinje cells and the degradation of dendrites are observed in the histopathological image of animals that received fluorine for a long period of time [79].

Neural cell adhesion molecules (NCAM) are membrane glycoproteins that are responsible for the adhesion and migration of cells of the nervous system, the development of axons and synapses and the activation of signal pathways [80]. The disturbances in the expression of the isoforms NCAM-120, NCAM-140 and NCAM-180 influence the cognitive functions of the nervous system, whereas the presence of NCAM-180 significantly influences the plasticity of neurons in the hippocampus. Nerve cells isolated from the hippocampus show a decreased expression of NCAM after incubation with fluorine (the applied fluorine concentrations, 20, 40 and 80 mg/L). The application of the 80-mg/L concentration resulted in a decrease in the amount of all of the three aforementioned isoforms, whereas with lower fluorine concentrations the expression of the isoform NCAM-180 also decreased significantly [40]. The decrease in the expression of NCAM influences the plasticity of neurons and causes disturbances in cognitive functions. This phenomenon is confirmed by experiments carried out on animals with the NCAM gene blocked, as they showed that the animals had problems with spatial learning [40, 81].

Animals exposed to fluorine suffered from chronic pain and had a higher sensitivity to pain. Furthermore, there was an increase in the expression of brain-derived neurotrophic factor (BDNF) and a decrease in the expression of GFAP in the cerebral cortex and hippocampus of those animals (the concentration of fluorine in drinking water, 50 and 150 mg/L) [30]. The expression of the BDNF is regulated, among others, by serine-threonine kinases, so the changes in the concentration of BDNF in nerve cells may be caused by the activation of proteins from the MAP kinase family by fluorine [4]. BDNF is a protein belonging to neurotrophins—a group of neurotrophic factors that includes substances supporting the differentiation and survivability of neurons [82]. BDNF regulates the growth and regeneration of neurons, and it influences their plasticity. The increase in its synthesis accompanies such processes as the damaging of tissue or ageing [83–86]. GFAP is a protein specific for astrocytes, and the changes in its expression influence the maturing of neurons and glial cells [87]. The increased concentration of BDNF in the nervous system with the simultaneous decrease in the concentration of GFAP indicates disorders in the maturing of nerve cells and the activation of repair processes aiming at the neutralization of the damage caused by the toxic influence of fluorine [11].

### The Influence of Fluorine on the Initiation of Apoptosis in the Central Nervous System

ROS in cells function as signalling particles and, in physiological concentrations, influence the activity of metabolic pathways. However, if their concentration in cells is too high, it leads to the oxidation of nucleic acids, including the oxidation of DNA and the braking of α-helix. The accumulation of many such changes in the DNA, detected by repair mechanisms, leads to the activation of the apoptosis pathway [88–90]. There was a significant increase in the number of apoptotic cells in the culture of nerve cells isolated from the hippocampus after 48 h of incubation with fluorine. The following concentrations of fluorine were used in the research—20, 40 and 80 mg/L. However, the increase in the concentrations of apoptotic cells appeared in the case of 40 and 80 mg/L [40].

Nuclear factor kappa B (NF-kB) is a transcription factor that participates in the processes related to cell growth, the regulation of cell cycle, the development of an inflammatory state and the response to stress [91–94]. Depending on its level of expression and the pathways it influences, it may protect cells from apoptosis or initiate the process [95, 96]. A research carried out on neurons isolated from the hippocampus of a rat incubated 24 h with fluorine (40 and 80 mg/L) indicated a significantly increased frequency of damage to the DNA and an increase in the synthesis of NF-kB [48]. Among animals exposed to lower concentrations of fluorine (30 mg/L), there was an increase in the expression of NF-kB which correlated with an increased concentration of calcium ions in the studied cells. It is widely known that fluorine increases the synthesis of ROS in neurons, which causes damage to the cell membrane. Calcium ions move through the damaged membrane to nerve cells causing, among others, an increase in the expression of NF-kB. Consequently, they influence the initiation of programmed death [95, 97].

The analyses carried out so far indicate that the apoptosis of neurons observed in chronic fluorosis may be activated by the mitochondrial pathway. It was proved that the processes related to nerve cell degeneration are influenced by MAP kinases, signal pathways with the participation of G proteins, calcium ions, the p38 protein and Jun N-terminal kinase (JNK) [98–100]. The 6-month exposure of rats to both low (5 mg/L) and high (50 mg/L) concentrations of fluorine in drinking water caused a significant increase in the number of apoptotic cells in the brain and in the content of the phosphorylated form of JNK. In the case of the total content of JNK, the changes were insignificant. The analysis suggests that fluorine stimulates apoptosis through the activation of JNK because in the brains of the exposed animals there is an increase in the content of the active (phosphorylated) form of this protein, and not its total content, in comparison to the animals from the control group [98]. JNK kinases influence apoptosis through the activation of caspases and the changes in the expression of genes associated with this process [101]. Furthermore, among adult rats supplied with fluorine in drinking water, there was an increased expression of the proapoptotic protein Bax and a decreased expression of the apoptosis inhibiting Bcl-2. The analysis carried out by means of the terminal deoxynucleotidyl transferase dUTP nick end labelling (TUNEL) method confirmed the increased intensity of apoptotic processes in brain structures of the animals (the concentration in drinking water, 60 and 120 mg/L) [60].

The results achieved in vivo were confirmed by in vitro studies. In the culture of cells of the SH-SY5Y line, after 48 h of incubation with fluorine (40 and 80 mg/L), there was an increase in the concentration of caspase-3 and in the expression of Fas, Fas-L, caspase-3 and caspase-8. The activity of caspase-3 is regulated by the changes in the expression and activity of other proapoptotic proteins, including caspase-8 and caspase-9 that are activated in the mitochondrial pathway. The damage of cells caused by the toxic influence of fluorine leads to the activation of the mitochondrial pathway of apoptosis through the activation of procaspase-8 by Fas and the activation of caspase-3 which eventually initiates the degradation of neurons [102].

### Summary

Previous studies on fluorine neurotoxicity showed that one of the main mechanisms that lead to the disturbances in central nervous system homeostasis is oxidative stress. ROS act as mediators in many processes and in high concentrations. They are able to initiate cell damage and metabolism disorders [38]. Fluorine is responsible for both—increase in ROS synthesis and lipid peroxidation and decrease in anti-oxidative enzyme activity in neurons and glia [43]. An excessive intake of fluoride is also responsible for the increase in AGE synthesis in CNS which leads to synthesis of transcription factors and proinflammatory substances including NF-kB, interleukins and MAP kinases [43]. Moreover, fluorine causes glial cell activation which is involved in inflammation in the brain and change in the expression of proteins which regulate neuron differentiation and proliferation and initiate apoptosis such as NCAM, GFAP, BDNF, JNK, Bax and Bcl2 [17, 30, 60, 61, 101]. The accumulation of this element in CNS causes cytological changes within neurons (changes in tubulin expression and concentration of Nissl bodies) and changes in neuron activity and their energy metabolism [1, 4, 9, 15, 30]. The accumulation of fluorine in the nervous system influences the synthesis of neurotransmitters, the activity of enzymes, the expression of receptors and the plasticity of neurons [105–107]. Numerous analyses carried out in in vivo and in vitro models confirmed that prolonged exposure to high concentrations of fluorine leads to the degeneration of neurons [1, 60].

The central nervous system during development is highly sensitive to the influence of fluorine due to its weakened protective mechanisms. In the childhood period, exposure to this element may cause permanent damage to the functions of all brain structures [103, 104]. Among both young and adult specimens exposed to the toxic influence of high doses of fluorine, we can observe impaired ability to learn, disturbances in memory and information processing and behavioural problems. All of these cause a decrease in the quality of life [42]. Numerous reports concerning the occurrence of endemic fluorosis lead to the establishment of an accepted concentration of fluorine in drinking water by the World Health Organization (WHO) at a level of which the element does not accumulate excessively in the human organism and does not cause adverse effects. The current value is set at 1.5 mg/L [1, 2]. However, recent findings concerning the toxic influence of this element on the nervous system, especially dangerous in relation to developing organisms, lead to higher restrictions in countries where fluorosis occurs frequently.
